# Mixed neuroendocrine-non-neuroendocrine neoplasms (MiNEN) in the non-ampullary region of the duodenum: a case report

**DOI:** 10.1093/jscr/rjaf189

**Published:** 2025-04-05

**Authors:** Kazuki Matsushita, Atsushi Urakami, Masaki Matsubara, Akihisa Akagi, Takashi Urano, Naomasa Ishida, Munenori Takaoka, Hideyo Fujiwara, Takashi Akiyama, Tomoki Yamatsuji

**Affiliations:** Department of General Surgery, Kawasaki Medical School General Medical Center, 2-6-1 Nakasange, Kita-ku, Okayama 700-8505, Japan; Department of General Surgery, Kawasaki Medical School General Medical Center, 2-6-1 Nakasange, Kita-ku, Okayama 700-8505, Japan; Department of General Surgery, Kawasaki Medical School General Medical Center, 2-6-1 Nakasange, Kita-ku, Okayama 700-8505, Japan; Department of General Surgery, Kawasaki Medical School General Medical Center, 2-6-1 Nakasange, Kita-ku, Okayama 700-8505, Japan; Department of General Surgery, Kawasaki Medical School General Medical Center, 2-6-1 Nakasange, Kita-ku, Okayama 700-8505, Japan; Department of General Surgery, Kawasaki Medical School General Medical Center, 2-6-1 Nakasange, Kita-ku, Okayama 700-8505, Japan; Department of General Surgery, Kawasaki Medical School General Medical Center, 2-6-1 Nakasange, Kita-ku, Okayama 700-8505, Japan; Department of Pathology, Kawasaki Medical School General Medical Center, 2-6-1 Nakasange, Kita-ku, Okayama 700-8505, Japan; Department of Pathology, Kawasaki Medical School General Medical Center, 2-6-1 Nakasange, Kita-ku, Okayama 700-8505, Japan; Department of General Surgery, Kawasaki Medical School General Medical Center, 2-6-1 Nakasange, Kita-ku, Okayama 700-8505, Japan

**Keywords:** mixed neuroendocrine-non-neuroendocrine neoplasm (MiNEN), neuroendocrine neoplasms (NENs), neuroendocrine carcinoma (NEC), duodenal tumor, duodenal cancer

## Abstract

Mixed neuroendocrine-non-neuroendocrine neoplasms (MiNENs), a very rare form of neuroendocrine neoplasm (NEN), are associated with poor prognosis. Herein, we present a rare case of duodenal MiNEN composed of neuroendocrine carcinoma (NEC) and adenocarcinoma in a non-ampullary lesion. A 70-year-old male referred to our hospital was found to have duodenal perforation with a tumor in the 2nd duodenal portion based on computed tomography. Biopsy revealed a poorly differentiated adenocarcinoma. Three weeks following emergency surgery for the perforation, pancreaticoduodenectomy of the duodenal tumor was performed. The resected specimen comprised a 45 × 35-mm protruding and circumferential tumor extracted from the 2nd portion, with no involvement of the papilla of Vater. Histopathological examination revealed coexisting poorly differentiated adenocarcinoma and NEC components. The final pathological diagnosis was MiNEN (por2 > sig + NEC). TNM: pT4b (SE), pN2, M0, pStageIIIb. The postoperative course was uneventful, developing peritoneal dissemination and multiple bone metastasis after 5 months, with death 7 months postoperatively.

## Introduction

Mixed neuroendocrine non-neuroendocrine neoplasm (MiNEN), a subtype of neuroendocrine neoplasm (NENs), is a heterogeneous tumor comprising both neuroendocrine carcinoma (NEC) and non-NEC components, each accounting for over 30% of tumor content [1]. MiNENs may arise throughout the gastrointestinal tract, but in small intestine they predominantly develop in the duodenum. Duodenal NENs, accounting for ~4% of all gastroenteropancreatic NENs, are mostly ampullary; non-ampullary duodenal MiNENs (duo-MiNENs) are extremely rare [2].

The biological features of MiNENs are mainly determined by the NEC component; however, compared to NECs, they are difficult to diagnose, and show a worse prognosis and more aggressive progression. Owing to the limited amount and quality of existing data, the pathology and optimal treatment course for duo-MiNENs remain unclear. Herein, we present a rare case of non-ampullary duo-MiNEN comprising NEC and adenocarcinoma.

## Case report

**Figure 1:**
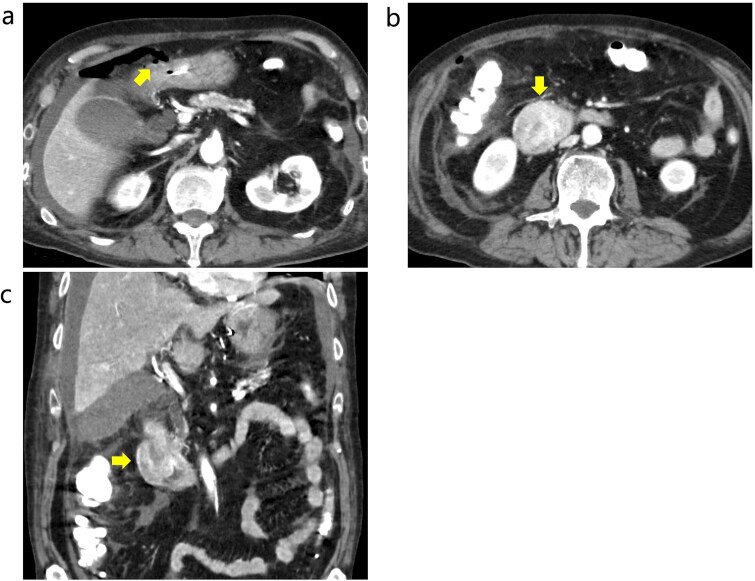
Contrast-enhanced CT before emergency surgery. (a) Free air was observed around the anterior wall in the duodenal bulb, and a perforation in the 1st portion was notes. The arrow indicates free air. (b and c) The tumor in the 2nd portion of duodenum was well enhanced. The arrows indicate the tumor.

**Figure 2:**
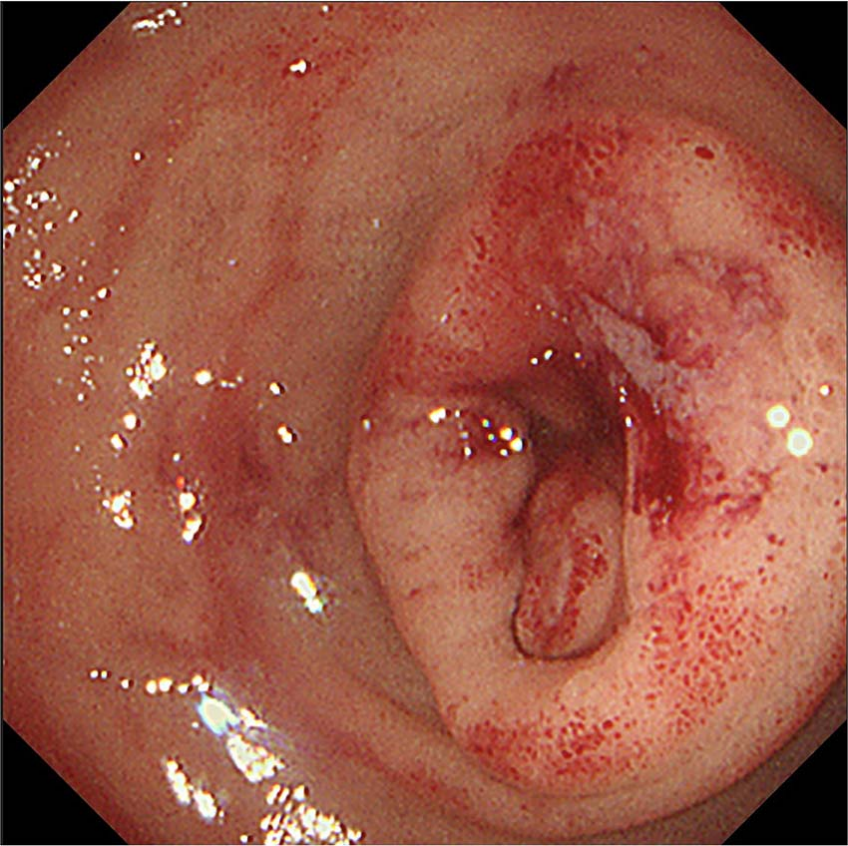
Gastro-duodenal endoscopy. Gastro-duodenal endoscopy revealed duodenal stenosis with the duodenal tumor in the 2nd portion. The tumor was biopsied, with subsequent analysis revealing adenocarcinoma.

**Figure 3:**
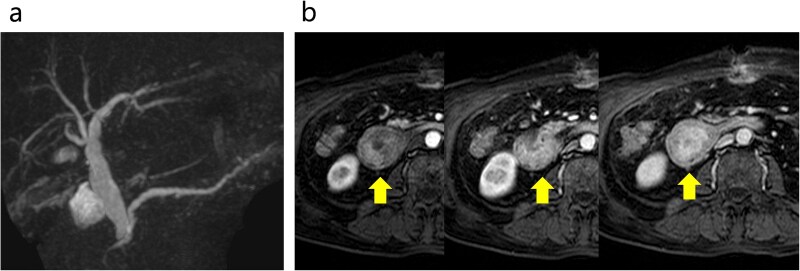
Abdominal MRI. (a) No stenosis was observed in the bile or pancreatic ducts. (b) Contrast enhanced MRI revealed early dark staining in the duodenal tumor. The arrows indicate the tumor.

A 70-year-old male who worked as a farmer following retirement from office-work was referred to our hospital with anorexia, nausea, and abdominal pain. The patient had a history of hypertension, diabetes mellitus, gastroesophageal reflux disease, and benign prostatic hyperplasia. He had not smoked for 10 years and consumed one glass of the alcoholic beverage shochu daily. Contrast-enhanced computed tomography (CT) of the abdomen revealed peritonitis/duodenal perforation and a 4 cm duodenal tumor in the 1st and 2nd duodenum portions, respectively (Fig. 1a–c). Emergency laparotomy was performed to close the perforation. Gastroduodenal endoscopy revealed duodenal stenosis with a tumor in the 2nd portion (Fig. 2). Biopsy indicated a poorly differentiated adenocarcinoma; however, magnetic resonance imaging (MRI) showed no stenosis in the bile or pancreatic ducts (Fig. 3a and b). Preoperative laboratory data revealed normal levels of the tumor markers carcinoembryonic antigen (CEA) 1.5 (<5.0) and CA19–9 12.6 (<37.0).

**Figure 4:**
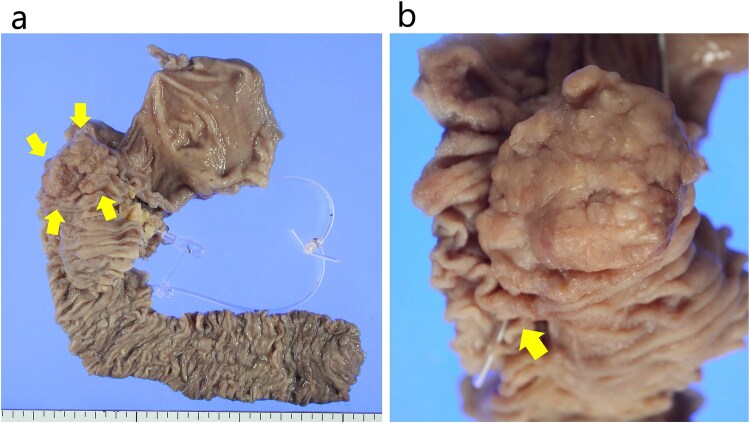
The resected specimen. (a) The specimen comprised a 45 × 35 mm sized protruded and circumferential tumor resected from the 2nd duodenal portion. The arrows indicate the tumor. (b) The papilla Vater was not involved in the tumor. The arrow indicates the papilla Vater.

**Figure 5:**
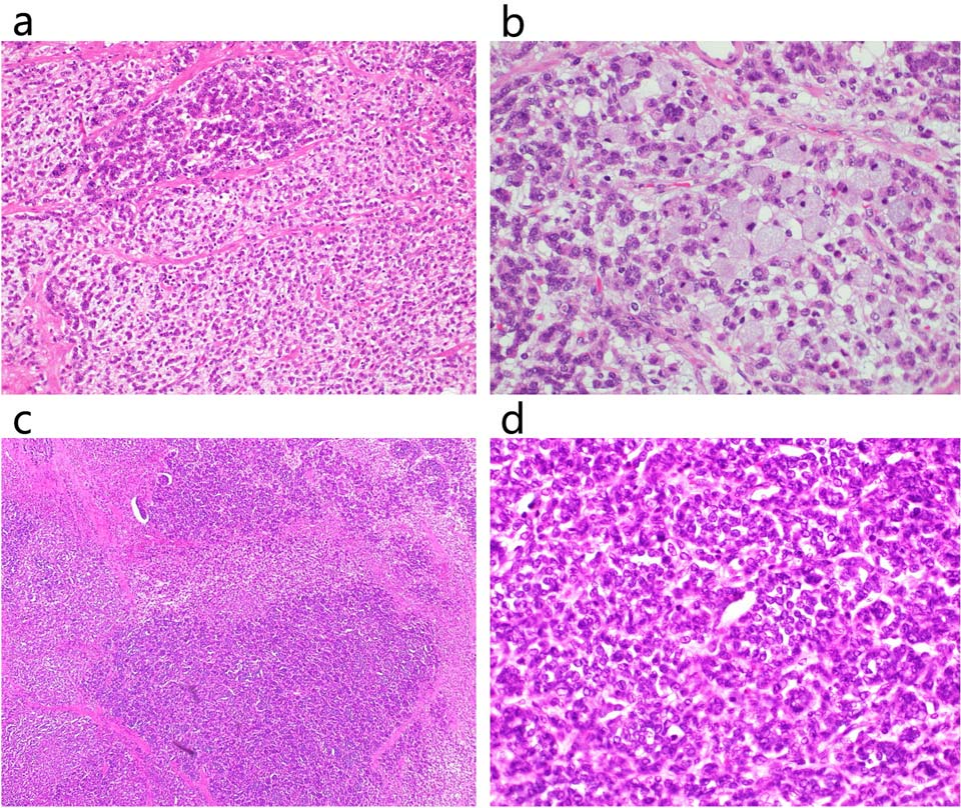
Histopathological examination. Histopathological examination revealed that the tumor had both adenocarcinoma and neuroendocrine components. (a) HE staining of a poorly differentiated adenocarcinoma region (×200). (b) HE staining of poorly differentiated adenocarcinoma and signet ring cell carcinoma (×400). (c) HE staining of NEC (×100). (d) HE staining of NEC (×400).

**Figure 6:**
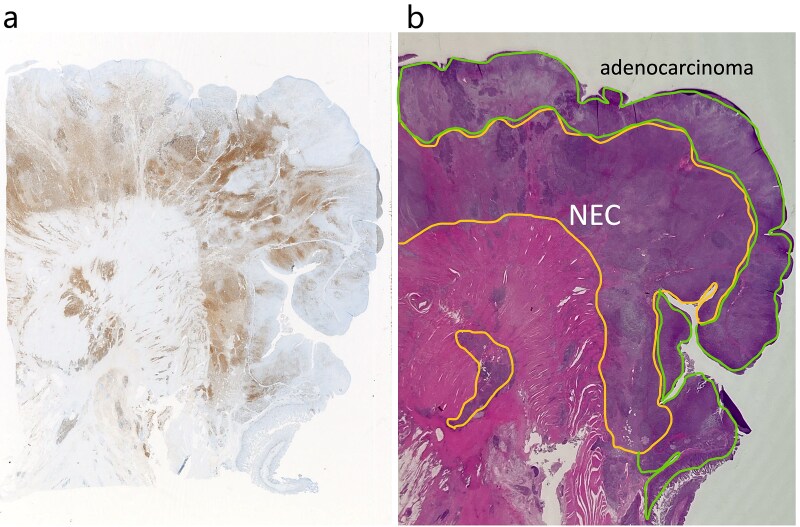
Adenocarcinoma and NEC components in the tumor. (a) Immunochemical staining of chromogranin a was positive in the NEC component (×20). (b) Adenocarcinoma and NEC components accounted for 60% was 40% of the tumor, respectively. Therefore, the tumor was diagnosed MiNEN (×20).

Three weeks post-operatively, the duodenal tumor was resected by pancreaticoduodenectomy (SSPPD-IIA-1) achieving R0 resection. The resected specimen comprised a 45 × 35-mm protruding and circumferential tumor in the 2nd portion with no involvement of the papilla of Vater (Fig. 4a and b). Histopathological examination revealed the coexistence of a poorly differentiated adenocarcinoma (Fig. 5a and b) and NEC (Fig. 5c and d). The tumor had invaded the pancreatic parenchyma and bile duct wall. Immunochemistry revealed positivity for the markers of tumoral neuroendocrine differentiation, CD56, synaptophysin, and chromogranin A. Genetic testing revealed negative results for the RAS and BRAF genes, and microsatellite instability. Adenocarcinoma and NEC components accounted for 60% and 40% of the tumor, respectively (Fig. 6a and b). Several regional lymph node metastases were observed. The final pathological diagnosis was MiNEN (por2 > sig + NEC), TNM pT4b (SE), INFc, ly1c, V1b, Pn1b, pN2, M0, and pStage IIb (T4bN2M0, AJCC/UICC 8th edition).

The patient’s postoperative course was uneventful; however, he refused chemotherapy, ultimately developing peritoneal dissemination and multiple bone metastases 5 months postoperatively, and dying 7 months postoperatively.

## Discussion

Herein, we present the clinical course of a rare case of non-ampullary duo-MiNEN comprising conventional adenocarcinoma and NEC. Duodenal NENs, a clinically rare entity, are mostly (66%–80%) low-grade, with over 90% arising in the first/second parts of the duodenum [2]. MiNENs in the ampullary region of the duodenum have been reported [3]; however, non-ampullary MiNENs are extremely rare.

Most MiNENs develop slowly and manifest with nonspecific clinical symptoms similar to traditional adenocarcinomas. The present patient presented with an unusual duodenal perforation caused by stenosis with the tumor. Duodenal perforation can develop in patients with NENs because of hypergastrinemia secondary to gastrinoma [3]. Concurrent cancer and gastroduodenal perforation has a high mortality rates and unfavorable short-term outcomes [4]; however, its rarity has led to a lack of published data on its emergent management.

With a short median survival of ˂12 months, MiNENs are generally associated with a worse prognosis. Poorly differentiated NECs, degree of adenocarcinoma differentiation, and NEC proportion severely affect the survival and prognosis of patients with MiNENs [5]. In one study of high-grade malignant MiNEN, an NEN component of >50% of the total tumor size was a poor prognostic factor [6]. In the present case, the adenocarcinoma and NEN components existed in a ratio of 60:40. A definitive diagnosis of MiNEN usually requires surgical resection. As the NEN component of an MiNEN is usually located in the deep submucosal layers, establishing a definitive diagnosis before resection is challenging [7]. In one systematic review, the accuracy of preoperative endoscopic diagnosis in 67 patients undergoing surgical resection for biliary MiNEN was 24.1% [8].

The latest guidelines from the European Neuroendocrine Tumor Society recommend considering surgical resection and the possibility of performing R0 for patients with localized resectable digestive NEC [9]. Adenocarcinoma- and NEC-driven cancers are commonly treated with a 5-FU based backbones and etoposide- and cisplatin-based therapies, respectively. Depending on size, non-ampullary duodenal NETs can be treated endoscopically or surgically [10]. Endoscopic resection is a safe, feasible option for non-ampullary duodenal NETs ≤10 mm in size without lymph node/distant metastasis [11]. However, guidelines for adjuvant/neoadjuvant chemotherapy for non-ampullary MiNENs are currently lacking. The metastatic status during diagnosis and histopathological tumor grading generally drive therapeutic choices. Moreover, many studies on digestive NEC do not distinguish between adjuvant, neoadjuvant, and perioperative chemotherapies, although pure neoadjuvant studies are rare [9]. Various studies have reported differing chemotherapeutic/surgical options for MiNEN, including carboplatin- and etoposide-based chemotherapy, followed by radical resection and postoperative adjuvant chemotherapy [12]; adjuvant chemotherapy to prevent recurrence [9, 13]; postoperative chemotherapy for improved survival [14]; and platinum-etoposide regimens [15]. Overall, for patients with localized digestive MiNENs, need for surgery and adjuvant chemotherapy [9] should be considered.

In conclusion, the rarity of this case and the unusual presentation of gastroduodenal perforation offer insights into the management and treatment of this condition. Further, the clinical outcomes underscore the importance of adjuvant chemotherapy.
